# Volatile Organic Compound Emissions from Prescribed Burning in Tallgrass Prairie Ecosystems

**DOI:** 10.3390/atmos10080464

**Published:** 2019

**Authors:** Andrew R. Whitehill, Ingrid George, Russell Long, Kirk R. Baker, Matthew Landis

**Affiliations:** 1 Office of Research and Development, U.S. Environmental Protection Agency, Research Triangle Park, NC 27709, USA; 2 Office of Air Quality Planning and Standards, U.S. Environmental Protection Agency, Research Triangle Park, NC 27709, USA

**Keywords:** prescribed fire, volatile organic compound, air pollution, tallgrass prairie, Flint Hills, TO-15, ozone, emissions

## Abstract

Prescribed pasture burning plays a critical role in ecosystem maintenance in tallgrass prairie ecosystems and may contribute to agricultural productivity but can also have negative impacts on air quality. Volatile organic compound (VOC) concentrations were measured immediately downwind of prescribed tallgrass prairie fires in the Flint Hills region of Kansas, United States. The VOC mixture is dominated by alkenes and oxygenated VOCs, which are highly reactive and can drive photochemical production of ozone downwind of the fires. The computed emission factors are comparable to those previous measured from pasture maintenance fires in Brazil. In addition to the emission of large amounts of particulate matter, hazardous air pollutants such as benzene and acrolein are emitted in significant amounts and could contribute to adverse health effects in exposed populations.

## 1. Introduction

Prescribed burning of grasslands plays a critical ecological and economic role in tallgrass prairie ecosystems, such as the Flint Hills range in Kansas, United States. Fire-adapted non-equilibrium tallgrass ecosystems benefit from the removal of standing dead vegetation leading to improved sun and water penetration of the soil, the recycling of nutrients such as nitrogen, and the killing of competing plants such as forbs and woody vegetation [1–3]. Extensive studies have looked at the impact of the timing and frequency of prescribed burns on factors such as species ecology, biodiversity, soil moisture and nitrogen cycling, net ecosystem carbon dioxide (CO_2_) exchange, air quality, and other factors [4–7]. In the absence of natural fire events, prescribed burning at least once every three years is necessary to prevent the permanent encroachment of woody plants and maintain the prairie ecosystem [5,8,9].

Springtime burning of tallgrass prairie can have a significant impact on local and regional air quality, with increased burning related to increases in fine particulate matter (PM_2.5_) [4,10] and ozone (O_3_) [4,11]. Emitted volatile organic compounds (VOCs) react in the troposphere to form O_3_ in the presence of nitrogen oxides (NO_x_), especially when meteorological conditions are favorable. VOCs emitted from biomass combustion are also precursors to reactive radical species that can drive secondary PM_2.5_ formation. O_3_ and PM_2.5_ are known to have negative human health impacts [12,13] and the population of the Flint Hills region have specific traits that make them more susceptible to impacts from smoke [14]. In addition to the criteria pollutants, fires are known sources of hazardous air pollutants, including formaldehyde, acrolein, and benzene. Measurements of grassland burning emissions of VOCs have been presented in the literature [15–26], but not for this ecoregion.

The goal of this study was to determine tallgrass prairie ecosystem-specific emission factors for 29 VOCs, including ten highly reactive alkenes, four toxic aromatic compounds (benzene, toluene, p-xylene, and ethylbenzene), and acrolein. Accurate estimates VOC emissions from prescribed fires in managed prairie ecosystems are critically important for air quality modeling systems to appropriately represent local and regional scale O_3_ and secondary PM_2.5_ formation [27,28]. Air quality models are needed to forecast smoke impacts and to replicate historical periods of smoke impacts to provide information about how best to balance air quality impacts and ecological goals related to grassland burning. Despite the significance of the prescribed grassland burning in the Flint Hills to local and regional air quality, ecosystem-specific emission factors for critical precursor species have not been measured. The results provided here will support future modeling of near field hazardous air pollutant exposure (e.g., acrolein and benzene), as well as the impact of prescribed burns on regional O_3_ and secondary organic aerosol production.

## 2. Materials and Methods

### 2.1. Sample Collection and Analysis

A series of gas phase species, including carbon monoxide (CO), CO_2_, and VOCs, were sampled at the Konza Prairie Long Term Ecological Research Site, downwind of prescribed prairie management fires. Konza Prairie is a well-characterized research site run by the Nature Conservancy and Kansas State University that is located in the Flint Hills region near Manhattan, KS, USA. The Konza Prairie has predominantly a native tallgrass prairie ecosystem and is divided into delineated research plots based on burning frequency and grazing type. We sampled during four burn days in March of 2017 (15 March 2017, 16 March 2017, 17 March 2017, and 20 March 2017).

On three of these sampling days (15 March 2017, 16 March 2017, and 20 March 2017), we collected a total of nine 6-L VOC canisters. Samples were collected in evacuated and cleaned SUMMA air sampling canisters (Andersen Samplers Inc., Atlanta, GA, USA and Scientific Instrumentation Specialists, Moscow, ID, USA) through a critical orifice to control collection rate. Canister pressure was measured immediately before sampling (to ensure vacuum) and after sampling. Canister integration times ranged from 30 min to 1 h and 2 min. All canister VOC data was normalized to concurrently measured carbon monoxide, so we do not expect the variation in canister integration times to affect the final emission factor or emission ratio calculations. CO, CO_2_, and CH_4_ were analyzed from the canister samples using gas chromatography. VOCs from canister samples were analyzed by gas chromatography mass spectrometry (GC-MS) following EPA Method TO-15 [29]. Further details on the analytical methods can be found in [30].

Continuous measurements of CO and CO_2_ were made using a Thermo Fisher Scientific (Franklin, Massachusetts, United Staets of America) Model 48c CO Analyzer and a California Analytical Instruments (CAI; Orange, CA, USA) Model 200 CO_2_ analyzer, both of which operate based on infrared absorption. Accounting for the manufacturer’s specified precision and accuracy, calibration uncertainty, and other factors, we expect continuous CO and CO_2_ measurement accuracy to be within ±10%. Multipoint calibrations produced coefficient of determination (*r*^2^) values ≥0.999 for CO and >0.990 for CO_2_.

Instruments were located inside a Chevrolet Suburban, with the sampling inlets located on top of the vehicle. Instruments were powered by a generator and a series of batteries located in a trailer towed behind the vehicle. During sampling, the Suburban was positioned downwind of the fire, and the trailer was located downwind of the Suburban, to allow sampling of fire emissions while limiting sampling of generator emissions. VOC canister samples were collected on top of the vehicle, next to the inlets for the continuous samplers.

**Figure 1.**
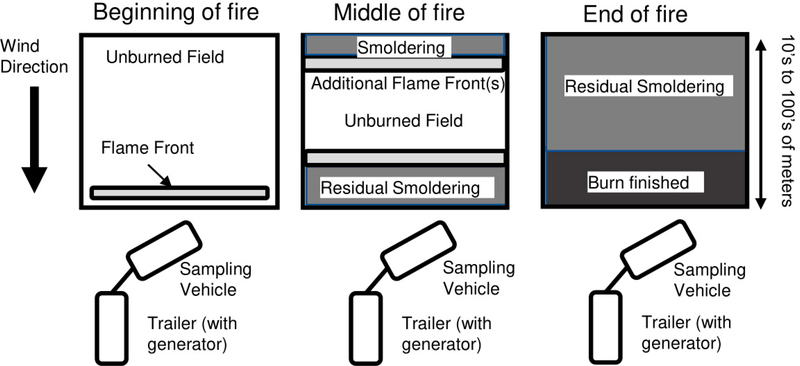
Diagram of sampling arrangement during burns, illustrating the relationship between the evolving flame front, the sampling trailer, and the generator relative to the wind direction.

During each burn, the gases sampled evolved from a nearby flaming front, usually a backfire, to lower emissions from residual smoldering combustion, with influence from an upwind headfire (and possibly flankfires) that were further away from the sampling vehicle. Towards the beginning of each burn there was a spike of emissions from a large flaming front, which was generally located within meters of the sampling vehicle. Within minutes, the flame front progressed tens of meters away from the vehicle and the vehicle was sampling emissions from an upwind flame front(s) mixed with residual smoldering emissions from the parts of the field that had already ceased flaming. Tens of minutes or longer into the burn (depending upon the plot size) the flame front had been exhausted or moved beyond where it could be sampled by the vehicle and the vehicle was sampling predominantly residual smoldering emissions. A diagram of the sampling setup during fire evolution is shown in Figure 1. In several cases, the vehicle was moved during sampling to remain within the progressing smoke plume. Estimated transit times between emission and sampling was in the order of 1 min (for sampling nearby emissions) to 10 min (for sampling emissions from further upwind).

### 2.2. Data Reduction and Calculations

We normalized the concentrations of emitted VOCs and carbon species to the major carbon species CO to determine the emission ratios:

(1) $${}^{\text{X}}\text{E}{\text{R}}_{\text{CO}}\text{=(ΔX/ΔCO}{\text{)}}_{\text{fire }},$$

where X is the species of interest and ΔX is the excess mixing ratio of species X, defined as

(2) $$\text{ΔX=}\;{\text{X}}_{\text{plume}}\;\text{−}\;{\text{X}}_{\text{background}}.$$

We computed emission ratios using regression analysis, computing the least squares linear regression of the species of interest with CO. We calculated regressions using both the ordinary least squares (OLS) model [31], and *a* regression through the origin (RTO) model [32], which forces the intercept term to be 0. Due to the small number of VOC samples (N = 9), we determine which model is appropriate using the corrected Akaike information criterion (AICc) [33].

We used calculated emission ratios to compute emission factors (grams of species X emitted per kilogram of biomass burned) based on the carbon mass balance method [34]. Big bluestem (Andropogon gerardii) is the dominant species in almost 90% of plots at Konza Prairie [35], and remains the dominant grass species over much of the Great Plains [36]. Elemental analysis of dried Andropogon gerardii is 49.1% carbon by weight [37], so there are 491 g of carbon for each kg of dry biomass, which was used along with emission ratios to compute emission factors.

We also calculated modified combustion efficiency (MCE) [38] based on Equation (3). MCE is an indicator of the amount of flaming versus smoldering combustion. To reduce the uncertainty from not precisely knowing the background CO_2_ and CO, we computed fire-averaged MCE from the ΔCO/ΔCO_2_ ratio, which we determined from regression analysis with the OLS model. MCEs for individual phases of the fire were calculated by assuming the lowest CO and CO_2_ value measured each day as the background concentration and calculating the excess mixing ratios of CO and CO_2_ using Equation (2). MCEs close to 1.0 are characteristic of flaming combustion, whereas MCEs of 0.6–0.8 are characteristic of the smoldering combustion.

(3) $$\text{MCE}\;=\;\Delta {\text{CO}}_{2}/\left(\Delta {\text{CO}}_{2}\;+\;\Delta \text{CO}\right).$$

Emission ratio and emission factor values are reported with 95% confidence intervals (95% CI).

## 3. Results

### 3.1. Major Carbon Species (CO, CO_2_, and CH_4_), and MCE

**Figure 2.**
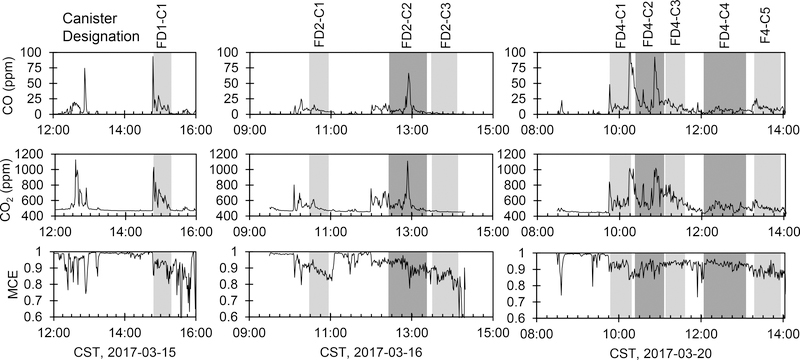
Continuous timeseries of CO, CO_2_, and MCE for the three days we sampled VOC canisters. Canister sampling periods are shaded and labeled with the canister designation above the CO timeseries.

Continuous timeseries of CO, CO_2_, and MCE are shown in Figure 2, with raw data provided in the Supplemental Information (Tables S1–S4). CO and CO_2_ were measured both in canisters and with continuous instruments, with linear regressions showing

(4) $$[\text{COcanister}]\;=\;0.82\cdot [\text{COcontinuous}]\;+\;1.24\;\mathrm{ppm}\;\left(r^{2}\;=\;0.981\right)$$

and

(5) $$\left[{\text{CO}}_{2},\text{canister}\right]\;=\;1.09\cdot \left[{\text{CO}}_{2},\text{continuous}\right]\;-\;18.69\;\text{ppm}\;\left(r^{2}\;=\;0.979\right).$$

Canister CO data was used for all canister (VOC) analyses because the canister CO reflects the integrated sample collected during canister sampling, including any inconsistencies in sampling rates or spatial heterogeneities between the canister inlet and the gas phase inlets. Other than the critical orifice, no additional flow controllers were used during the vacuum canister sampling to modulate sampling flow rates. The minimum continuous CO concentration from each sampling day was used as the “background” CO concentration when calculating the excess mixing ratios of CO for the canister samples. Background values were 0.32, 0.27, and 0.06 ppm for 15 March 2017, 16 March 2017, and 20 March 2017, respectively.

**Figure 3.**
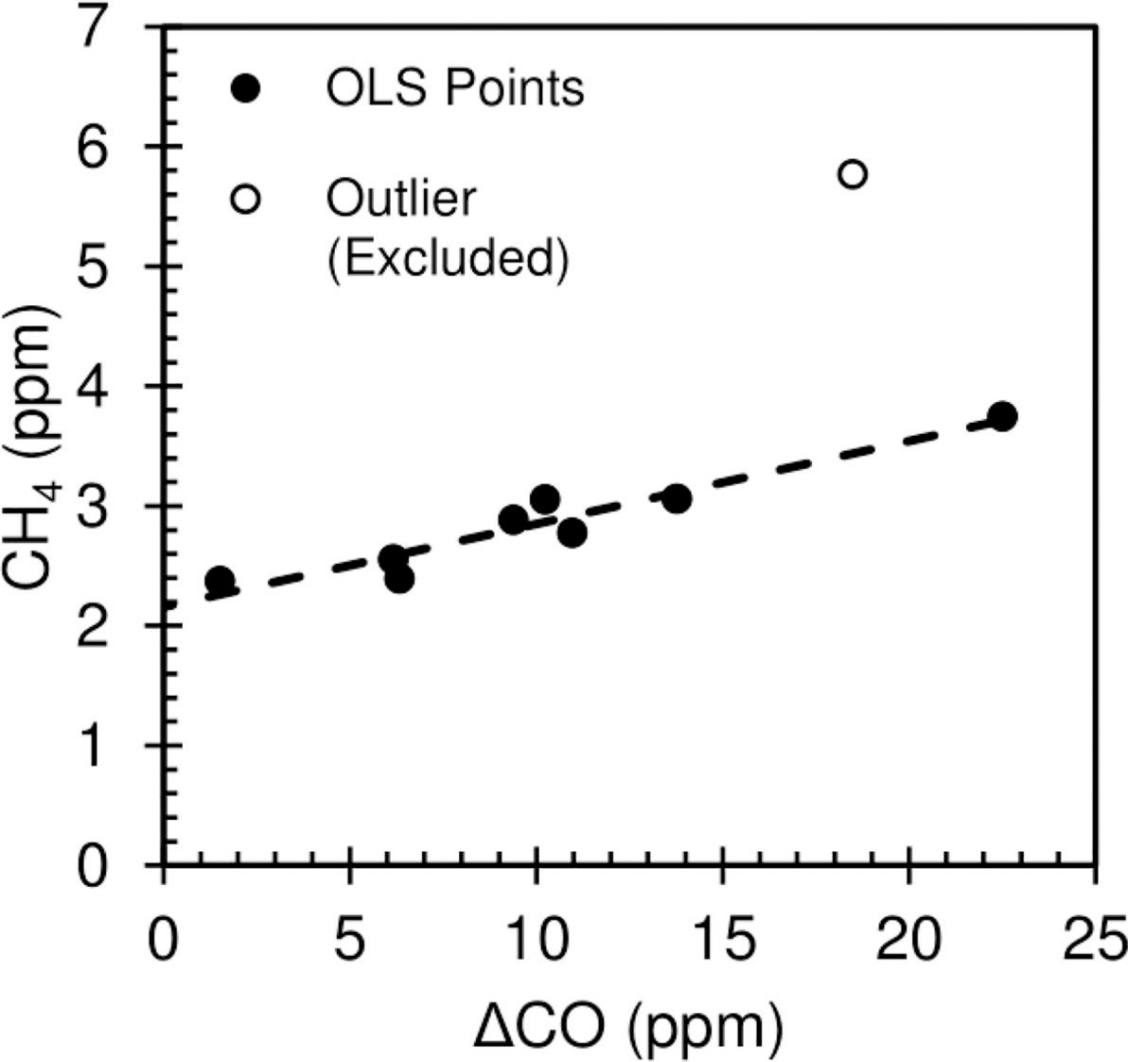
CH_4_ vs ΔCO for the nine VOC canister samples, showing the eight fit points (with the ordinary least squares regression line) and the one outlier point.

Methane (CH_4_) emission ratios (relative to CO from the canisters) and background values were determined using an ordinary least squares linear regression of ΔCO versus CH_4_. Of the 9 points, 8 lay on a line and the ninth (FD4-C1) is a high outlier (Figure 3). We hypothesize there to be an additional source of methane contributing to FD4-C1. Therefore, we calculated the regression of ΔCO versus CH_4_ for the other 8 samples and excluded the outlier sample from analysis. The OLS regression gave a slope (^CH4^ER_co_) value of 0.0693 (0.0485, 0.0902, 95% CI) and an intercept (background CH_4_) of 2.1558 ppm (1.9123, 2.3993). Therefore, our best estimate of the ^CH4^ER_co_ = 0.0693, with a 95% confidence interval of (0.0485, 0.0902). Raw methane concentrations from each canister are given in Table S5. VOC emission ratios are discussed in Section 3.2.

### 3.2. Volatile Organic Compound Emission Ratios

Analysis of the VOC canister samples identified 32 compounds present above the method quantification limit (MQL, 3x method detection limit or MDL ) in at least 8 of the 9 samples. Compounds present above the MQL in only 8 samples were below the MQL in FD2-C3, which had the lowest CO concentrations. On the basis of the AICc criterion and the intercept term of the OLS model (Table S6), we determined the RTO model to be the better choice to explain the relationship between ΔVOC and ΔCO for all but 3 of these compounds. Of these 3 compounds, two of them (dichlorodifluoromethane, and trichlorofluoromethane) were present in similar concentrations in all samples and seem to originate from the background air and only emitted in negligible amounts by the fire. The final compound (chloromethane) had likely had contributions from both the background air and the fire. The OLS model for chloromethane estimated *a* ^CH3C1^ER_co_ value of 0.074 (−0.002, 0.150) 10^−3^ with art intercept of 0.957 (−0.008,1.922) 10^−3^ ppm, so the background (intercept) term dominates the measured concentrations.

The remaining 29 compounds include unsaturated C3 (propylene), C4 (1-butene, cis-2-butene, trans-2-butene, and 1,3-butadiene), C5 (1-pentene, cis-2-pentene, trans-2-pentene, isoprene), and some C6 (1-hexene) compounds—alkanes (propane, butane, isopentane, n-pentane, n-hexane), simple nitriles (acetonitrile, acrylonitrile), simple aromatic compounds (benzene, toluene, ethylbenzene, and p-xylene), as well as several oxygenated VOCs (acrolein, ethanol, acetone, vinyl acetate, and 2-butanone). Emission factors for the unsaturated compounds (alkenes) were higher than emission factors of corresponding saturated compounds. The four measured species with the highest emission ratios were propylene, acetonitrile, acrolein, and acetone.

Table S5 provides measured concentrations for all 119 targeted VOCs (as well as CO, CO_2_, and CH_4_) alongside MDL values. 55 of these compounds were not detected or below the MQL for all 9 canister samples, with most of these compounds being halogenated compounds. The three halogenated compounds (dichlorodifluoromethane, trichlorofluoromethane, and chloromethane) measured in all samples were poorly correlated with CO, suggesting that they were likely present in background air, although chloromethane showed some evidence of emission for the prescribed fires. Compounds present in some, but not all, samples tended to be branched alkanes. These species did not provide sufficient data to estimate the emission factors, so we provide only the measured concentrations for these species.

### 3.3. VOC Emission Factors for Tallgrass Prairie Burns

Continuous CO and CO_2_ data from all four fire days was combined and regression analysis was used to estimate the study averaged ΔCO/ΔCO_2_ value of 0.10. We assumed this was the approximate value for the entire study period, although our ground measurements may be biased towards residual smoldering combustion compared to aircraft and aerostat measurements [17], and thus have a lower MCE than the fire as a whole. Combining this value with emission ratios of methane (^CH4^ER_co_ = 0.0693) and VOC carbon (^C-VOC^ER_co_ = 0.096, based on measured VOC concentrations), we estimate the average product distribution of gaseous carbon from the fire plumes is approximately 89.56% CO_2_, 8.96% CO, 0.62% CH_4_, and 0.86% other speciated VOCs. From this, we can calculate the emission factors of each emitted carbon species (Table 1), assuming an average ΔCO/ΔCO2 value of 0.10 (MCE = 0.91). Given the average biomass density of tallgrass prairies (4220 kg of biomass per hectare) [39], we also estimate the total emissions of each species per hectare burned.

## 4. Discussion

### 4.1. Comparison with Literature Emission Factors

Emission factors measured here are compared with values reported in [40] and [41] for different ecosystem categories. The best correspondence between measured carbon (CO, CO_2_, CH_4_, and VOC) emissions factors and literature values for similar ecosystems comes from the “Pasture Maintenance” fires in [41], which is a summary of ground and aircraft measurements of emission factors from pasture maintenance fires in Brazil [18,23,25]. Although the source measurements were mostly made by aircraft, the emission factors were scaled to account for a larger impact of residual smoldering combustion for near-surface emissions [41]. The Brazilian pasture ecosystem, which consists of managed grazing grasslands with small shrubby material, is similar to the central Kansas tallgrass prairies. However, the Brazilian fires may contain a higher portion of residual woody debris from former forests in the region versus the regularly burned grasslands at Konza, which had limited woody material.

**Figure 4.**
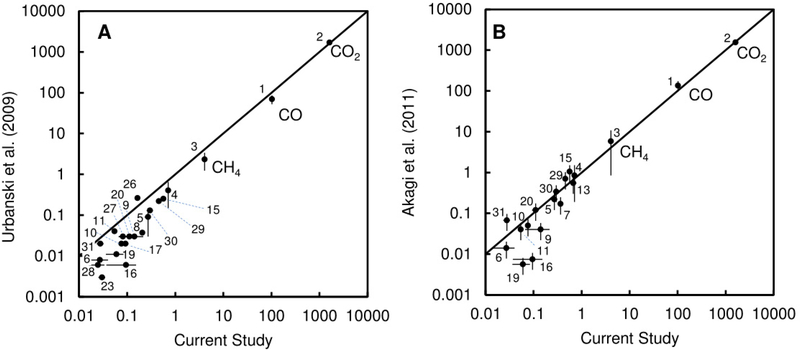
(**A**) Comparison of emission factors for CO, CO_2_, and VOCs obtained in the current study with those reported by [40]; and (**B**) comparison of emission factors for CO, CO_2_, and VOCs obtained in the current study with those reported by [41]. Points are labelled with numbers that correspond with the Species Number in Table 1.

The emission factors obtained in the present study and those originated from [41] (pasture burning category) are shown in Figure 4B. Emission factors for four hydrocarbons (butane, iso-butane, n-pentane, and iso-pentane) were higher in the present study than in the tabulated values of [41]. In the present study, these species showed weaker correlation with CO (with *r*_Pearson_ values of 0.532, 0.580, 0.559, and 0.676) than all the other nonhalogenated species (*r*p_earson_ > 0.7) except tert-butanol (*r*p_earson_ = 0.692). Therefore, it is possible that our estimates for these species are biased by an alternative source in some, but not all, of the canister samples. Accidental sampling of a hydrocarbon fuel source (such as gasoline or kerosene) in some of the samples could cause an overestimate of emission factors for these species. Possible sources include gasoline from the vehicles used by the fire teams and fumes from the drip torches used to start the burns. Ethyltoluene estimates from the “pasture burning” category of [41] are higher than the values we measured here. The tabulated values of [41] provide a good estimate for VOC emission factors of species not measured in the present study for modeling biomass burning emissions from tallgrass prairie ecosystems.

Our emission factors were higher than the “temperate rangeland” emission factors in [40] (Figure 4A). The [40] estimates are based mostly on airborne measurements, whereas our measurements are leased on ground-level emissions. This is reflected in the higher MCE (0.939) in [40] versus the present study Our estimates of VOCs may be biased by the influence of smoldering combustion on the near-ground measurements compared to the integrated fire emissions (near-ground and airborne). Comparisons of aircraft and ground-based emissions measurements from forests show that ground-based measurements of residual smoldering combustion can produce higher emission factors for VOCs and can have a significant impact on total-fire emissions of many VOC species [42]. There is also a significant sampling bias in how we sample the plumes from the ground, and what we measure as an “average” MCE may not reflect the MCE from integrated measurements of the entire fire. It is likely that a combination of both ground-based and aircraft data (or the use of tracer compounds) is necessary to fully quantify the MCE and emissions profiles from the integrated fire.

### 4.2. VOC Reactivity in Fresh Plumes

Prescribed fires in the Flint Hills region can have a significant impact on regional ozone measurements during the early spring burn period. Measured ozone values are higher on years when more acres are burned, with statistical modeling suggesting increases in 8-h ozone values of 12–30 ppb that can be attributed to the fires [11]. It has been documented that high VOC concentrations from wildfires, diesel vehicles, and fireworks can cause positive interferences in ultraviolet photometric ozone monitors, such as those used at many regulatory monitoring sites [43–46]. Prescribed burning in the area has directly contributed to regulatory exceedances of the ozone National Ambient Air Quality Standards in Wichita and Kansas City, two urban areas highly impacted by prescribed fires in the Flint Hills [47].

Photochemical modeling of fire chemistry using the Community Multiscale Air Quality Modeling System (CMAQ) suggests prescribed burning contributions to ozone on a regional level but overestimate ozone concentrations at fire-impacted monitoring sites [27]. Although some of the model overestimates were attributed to radiative feedbacks (i.e., the impact of aerosols on photolysis rates), it was unable to account for the entire issue. The authors of [27] identified the reactivity of the VOC mixture as potentially being a critical parameter in understanding the downwind impacts of prescribed fires in the region. This is particularly important with respect to secondary production of formaldehyde, acetaldehyde, and higher aldehydes, which are important precursors of HOx radical production and drive O3 production.

**Figure 5.**
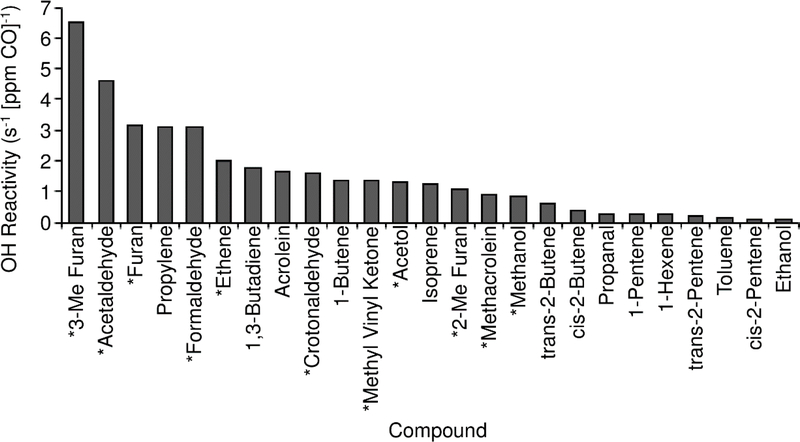
OH reactivity of 25 highest reactivity VOCs measured or estimated in this study. Compounds marked with a * are estimated from the “Pasture Maintenance” category [41].

OH reactivity of the measured VOC compounds can be determined from the measured concentrations (or emission ratios) and the rate factor of each compound with OH [48]. VOC OH reactivity is dominated by the short chain (C2 + C3) alkenes, as well as isoprene and acrolein. Propylene dominates the OH reactivity of the measured compounds (26.8%), with 1,3-butadiene (15.4%), acrolein (14.8%), and 1-butene (12.2%) also accounting for significant portions. Combined with isoprene (10.8%) and the 2-butene isomers (8.5%), these compounds account for 88.5% of the OH reactivity of measured compounds. The total OH reactivity of the measured VOCs is 11.4 s^−1^ [ppm CO]^−1^. Assuming similar emission factors to the “Pasture Maintenance” fires from [41] (see Section 4.2), we can estimate emission ratios and contribution to reactivity from unmeasured compounds. We used the emission factor values for ethene, methanol, acetol, and several reported ketones, aldehydes, and furans from [41] to compute the estimated contribution of those compounds to VOC OH reactivity as well. Of these compounds, ethene (2.0 s^−1^ [ppm CO]^−1^), formaldehyde (3.1 s^−1^ [ppm CO]^−1^), acetaldehyde (4.6 s^−1^ [ppm CO]^−1^), furan (3.1 s^−1^ [ppm CO]^−1^), and 3-methyl furan (6.5 s^−1^ [ppm CO]^−1^) contributed the most to VOC OH reactivity. Total VOC OH reactivity of the measured and estimated compounds is 38.2 s^−1^ [ppm CO]^−1^. Contributions to OH reactivity from individual measured or estimated species are shown in Figure 5.

Comprehensive laboratory-based studies using chemical ionization mass spectrometry to characterize OH reactivity [49,50] show that VOC OH reactivity is dominated by furans, other oxygenated VOCs, alkenes, and aromatic compounds. Although these studies have focused primarily on woody fuels, they reveal that there is a considerable amount of OH reactivity contributed by compounds not measured or estimated for this ecosystem (or similar ecosystems). The addition of functionalized and polyfunctionalized compounds in [50] compared to [49] had a significant effect on total OH reactivity, as many of these compounds are more reactive than the simple aldehydes, furans, alkenes, and aromatics measured here. Therefore, our estimate of OH reactivity should be considered a lower-bound, and additional studies using high-resolution chemical ionization mass spectrometry should be considered to more fully characterize emissions from grassland and pasture ecosystems.

Understanding ozone formation from biomass burning must go beyond measuring emission factors near source (or in the laboratory) and must also consider plume evolution. The first several hours of plume evolution are critical to modeling fire chemistry and associated ozone production. Measurements of OH concentrations in biomass burning plumes suggest levels in the order of 1 × 10^7^ molecules cm^−3^ within the first 20 to 40 min [24], which are similar to the values of 1.5 × 10^7^ molecules cm^−3^ to 1.9 × 10^7^ molecules cm^−3^ estimated from measuring the decrease of VOC species during plume aging [20]. In both cases, these values are significantly higher than background values, driving photochemical reactions at a faster rate than under typical ambient conditions. Oxygenated compounds, including both species we measured (acetone, ethanol) and many species we did not measure (such as formaldehyde, formic acid, methanol, and others) can have a significant impact on plume chemistry during the first several hours postemission, driving rand ozone production [51]. More complex oxygenated VOCs (and polyfunctionalized compounds) have more complicated chemistry and contribute significantly to both OH reactivity and potential secondary organic aerosol formation [49,50]. Secondary production of many oxygenated compounds is also relatively fast, with some fires showing formaldehyde and methanol concentrations increasing within hours of emission [52]. Burning in the Flint Hills is typically conducted during the daytime on sunny days with limited wind. Thus, due to both burn restrictions and for convenience issues, burns are most likely to occur on days when the conditions are ideal for rapid photochemistry and intense ozone production.

## 5. Conclusions

We measured ground-based VOC emission factors from prescribed fires in the Tallgrass Prairie ecosystem of the central United States. This study is the first reported VOC emission factors for this particular ecoregion, despite regular prescribed burning being implicated in air quality issues downwind. Regular prescribed burning of the tallgrass prairie is essential for ecosystem maintenance and is considered beneficial for agribusiness, but proper burn management. must be practiced to minimize regional air quality impacts. Improved constraints on emissions of primary air pollutants (and pollutant precursors) such as VOCs are critical to enhancing the performance of deterministic models of downwind air quality impacts, which is essential to proper planning and management of the prescribed fires. Despite the significance of prescribed burning to both agriculture and health, there is limited data about emission factors for tallgrass prairie ecosystems. We provide ground-based emission factors for a series of 29 reactive VOCs that are directly applicable to prescribed burns in the tallgrass prairie ecosystems. Our measured VOC emissions are dominated by reactive alkenes and oxygenated organic compounds, and the emission factors are similar to those previously measured in pasture fires in Brazil. The “Pasture Maintenance” values from [41] are recommended for estimating emission factors of species not measured in this study.

## Supplementary Material

Supplement1

## Figures and Tables

**Table 1.** Estimated emission factors (in grams of species per kilogram of dry biomass).

Species (Name)	Species Number	Emission Factor (g/kg)	95% CI (g/kg)	10^3^·^X^ER_CO_	95% CI	Emissions (kg/ha)
Carbon Monoxide	1	102.6		1		432.81
Carbon Dioxide	2	1611.5				6800.36
Methane	3	4.070	(2.848,5.298)	69.3	(48.5,90.2)	17.18
Propylene	4	0.713	(0.654,0.773)	4.630	(4.242,5.018)	3.01
Propane	5	0.270	(0.230,0.310)	1.672	(1.426,1.918)	1.14
Isobutane	6	0.027	(0.014,0.040)	0.127	(0.065,0.189)	0.11
1-Butene	7	0.363	(0.328,0.398)	1.767	(1.598,1.936)	1.53
1,3-Butadiene	8	0.207	(0.187,0.227)	1.046	(0.947,1.146)	0.87
Butane	9	0.141	(0.066,0.216)	0.662	(0.311,1.014)	0.59
trans-2-butene	10	0.076	(0.067,0.085)	0.370	(0.328,0.413)	0.32
cis-2-butene	11	0.054	(0.048,0.061)	0.264	(0.232,0.295)	0.23
Ethanol	12	0.160	(0.114,0.206)	0.949	(0.677,1.220)	0.68
Acetonitrile	13	0.669	(0.559,0.779)	4.452	(3.720,5.184)	2.82
Acrolein	14	0.704	(0.628,0.780)	3.431	(3.061,3.802)	2.97
Acetone	15	0.566	(0.520,0.613)	2.663	(2.443,2.882)	2.39
iso-Pentane	16	0.095	(0.037,0.153)	0.359	(0.140,0.577)	0.40
1-Pentene	17	0.093	(0.079,0.106)	0.361	(0.309,0.412)	0.39
Acrylonitrile	18	0.094	(0.082,0.106)	0.482	(0.421,0.543)	0.40
n-Pentane	19	0.060	(0.036,0.083)	0.226	(0.136,0.316)	0.25
Isoprene	20	0.111	(0.093,0.128)	0.486	(0.410,0.562)	0.47
trans-2-pentene	21	0.030	(0.025,0.035)	0.117	(0.098,0.135)	0.13
cis-2-pentene	22	0.017	(0.014,0.019)	0.065	(0.056,0.073)	0.07
Tert-Butanol	23	0.030	(0.019,0.041)	0.111	(0.071,0.151)	0.13
Cyclopentane	24	0.012	(0.008,0.016)	0.047	(0.032,0.062)	0.05
Vinyl Acetate	25	0.324	(0.260,0.389)	1.029	(0.824,1.233)	1.37
2-Butanone	26	0.164	(0.144,0.185)	0.622	(0.544,0.699)	0.69
1-Hexene	27	0.081	(0.069,0.093)	0.263	(0.223,0.302)	0.34
n-Hexane	28	0.025	(0.016,0.033)	0.078	(0.050,0.106)	0.10
Benzene	29	0.457	(0.439,0.475)	1.596	(1.533,1.660)	1.93
Toluene	30	0.297	(0.253,0.341)	0.880	(0.749,1.011)	1.25
Ethylbenzene	31	0.028	(0.023,0.032)	0.071	(0.060,0.083)	0.12
p-Xylene	32	0.021	(0.016,0.027)	0.055	(0.041,0.069)	0.09
