# Supplementary material for: Volatile Organic Compound Emissions from Prescribed Burning in Tallgrass Prairie Ecosystems
Source: Atmosphere (Basel). Author manuscript; Available in PMC 2020 Jan 1. (PMC6781241; doi:10.3390/atmos10080464)
Supplement: Supplement1 [file NIHMS1539427-supplement-Supplement1.zip › DataDictionary.docx]

**Dataset Name:**

Volatile organic compound emissions from prescribed burning in tallgrass prairie ecosystems

Updated 2019-06-18, Andrew Whitehill

**TableS1.csv**

*File Name:*
TableS1.csv

*Description:*
Continuous monitor data from 2017-03-15 burns

*Columns:*
**CST** – Date and time in Central Standard Time (CST), in “YYYY-mm-dd HH:MM” format
**CO_ppm** – Carbon monoxide (CO) concentration, in parts per million volume (ppm)
**CO2_ppm** – Carbon dioxide (CO2) concentration, in parts per million volume (ppm)
**MCE** – Modified combustion efficiency

**TableS2.csv**

*File Name:*
TableS2.csv

*Description:*
Continuous monitor data from 2017-03-16 burns

*Columns:*
**CST** – Date and time in Central Standard Time (CST), in “YYYY-mm-dd HH:MM” format
**CO_ppm** – Carbon monoxide (CO) concentration, in parts per million volume (ppm)
**CO2_ppm** – Carbon dioxide (CO2) concentration, in parts per million volume (ppm)
**MCE** – Modified combustion efficiency

**TableS3.csv**

*File Name:*
TableS3.csv

*Description:*
Continuous monitor data from 2017-03-17 burns

*Columns:*
**CST** – Date and time in Central Standard Time (CST), in “YYYY-mm-dd HH:MM” format
**CO_ppm** – Carbon monoxide (CO) concentration, in parts per million volume (ppm)
**CO2_ppm** – Carbon dioxide (CO2) concentration, in parts per million volume (ppm)
**MCE** – Modified combustion efficiency

**TableS4.csv**

*File Name:*
TableS4.csv

*Description:*
Continuous monitor data from 2017-03-20 burns

*Columns:*
**CST** – Date and time in Central Standard Time (CST), in “YYYY-mm-dd HH:MM” format
**CO_ppm** – Carbon monoxide (CO) concentration, in parts per million volume (ppm)
**CO2_ppm** – Carbon dioxide (CO2) concentration, in parts per million volume (ppm)
**MCE** – Modified combustion efficiency

**TableS5.csv**

*File Name:*
TableS5.csv

*Description:*
Raw VOC canister data

*Columns:*
**“” <first column>** – Name of species or parameter
**Units** – Units or format for species or parameter, ppb = parts per billion volume, ppm = parts per million volume
**MDL-GCMS** – Method detection limit (MDL) for the GC-MS analysis (excluding other analytical steps)
**FD1-C1** – Raw data from Fire Day 1 (2017-03-15), Canister #1
**FD2-C1** – Raw data from Fire Day 2 (2017-03-16), Canister #1
**FD2-C2** – Raw data from Fire Day 2 (2017-03-16), Canister #2
**FD2-C3** – Raw data from Fire Day 2 (2017-03-16), Canister #3
**FD4-C1** – Raw data from Fire Day 4 (2017-03-20), Canister #1
**FD4-C2** – Raw data from Fire Day 4 (2017-03-20), Canister #2
**FD4-C3** – Raw data from Fire Day 4 (2017-03-20), Canister #3
**FD4-C4** – Raw data from Fire Day 4 (2017-03-20), Canister #4
**FD4-C5** – Raw data from Fire Day 4 (2017-03-20), Canister #5
**MQL(FD1-C1)** – Method detection limit for Fire Day 1 (2017-03-15), Canister #1
**MQL(FD2-C1)** – Method detection limit for Fire Day 2 (2017-03-16), Canister #1
**MQL(FD2-C2)** – Method detection limit for Fire Day 2 (2017-03-16), Canister #2
**MQL(FD2-C3)** – Method detection limit for Fire Day 2 (2017-03-16), Canister #3
**MQL(FD4-C1)** – Method detection limit for Fire Day 4 (2017-03-20), Canister #1
**MQL(FD4-C2)** – Method detection limit for Fire Day 4 (2017-03-20), Canister #2
**MQL(FD4-C3)** – Method detection limit for Fire Day 4 (2017-03-20), Canister #3
**MQL(FD4-C4)** – Method detection limit for Fire Day 4 (2017-03-20), Canister #4
**MQL(FD4-C5)** – Method detection limit for Fire Day 4 (2017-03-20), Canister #5

**TableS6.csv**

*File Name:*
TableS6.csv

*Description:*
Regression statistics for VOC samples

*Columns:*
**Name** – Species whose concentration is regressed against carbon monoxide
**OLS.slope** – Slope estimate from ordinary least squares (OLS) regression
**OLS.slope.SE** – Standard error of slope estimate from ordinary least squares (OLS) regression
**OLS.slope.t** – t value for slope estimate from ordinary least squares (OLS) regression
**OLS.slope.Pr** – p value for slope estimate from ordinary least squares (OLS) regression
**OLS.slope.025** – lower bound of 95% confidence interval for slope estimate from ordinary least squares (OLS) regression
**OLS.slope.975** – upper bound of 95% confidence interval for slope estimate from ordinary least squares (OLS) regression
**OLS.intercept** – intercept estimate from ordinary least squares (OLS) regression
**OLS.intercept.SE** – standard error of intercept estimate from ordinary least squares (OLS) regression
**OLS.intercept.t** – t value for intercept estimate from ordinary least squares (OLS) regression
**OLS.intercept.Pr** – p value for intercept estimate from ordinary least squares (OLS) regression
**OLS.intercept.025** – lower bound of 95% confidence interval for intercept estimate from ordinary least squares (OLS) regression
**OLS.intercept.975** – upper bound of 95% confidence interval for intercept estimate from ordinary least squares (OLS) regression
**OLS.r.squared** – Coefficient of determination (r^2^ or R^2^) from ordinary least squares (OLS) regression
**OLS.r.squared.adj** – Adjusted coefficient of determination (r^2^ or R^2^) from ordinary least squares (OLS) regression
**OLS.SSE** – Sum of squared errors (SSE) from ordinary least squares (OLS) regression
**RTO.slope** – Slope estimate from regression through origin (RTO) regression
**RTO.slope.SE** – Standard error of slope estimate from regression through origin (RTO) regression
**RTO.slope.t** – t value for slope estimate from regression through origin (RTO) regression
**RTO.slope.Pr** – p value for slope estimate from regression through origin (RTO) regression
**RTO.slope.025** – lower bound of 95% confidence interval for slope estimate from regression through origin (RTO) regression
**RTO.slope.975** – lower bound of 95% confidence interval for slope estimate from regression through origin (RTO) regression
**RTO.r.squared** – Coefficient of determination (R^2^) from regression through origin (RTO) regression
**RTO.r.squared.adj** – Adjusted coefficient of determination (R^2^) from regression through origin (RTO) regression
**RTO.SSE** – Sum of squared errors (SSE) from regression through origin (RTO) regression
**OLS.AIC** – Akaike information criterion (AIC) for ordinary least squares (OLS) regression
**RTO.AIC** – Akaike information criterion (AIC) for regression through origin (RTO) regression
**OLS.AICc** – Corrected Akaike information criterion (AIC) for ordinary least squares (OLS) regression
**RTO.AICc** – Corrected Akaike information criterion (AIC) for regression through origin (RTO) regression
**N** – Number of samples used in regression analysis
**r_pearson** – Pearson product-moment correlation coefficient
